# TNF-α May Exert Different Antitumor Effects in Response to Radioactive Iodine Therapy in Papillary Thyroid Cancer with/without Autoimmune Thyroiditis

**DOI:** 10.3390/cancers13143609

**Published:** 2021-07-19

**Authors:** Dan Cristian Gheorghe, Marcel Marian Stanciu, Anca Zamfirescu, Adina Elena Stanciu

**Affiliations:** 1 ENT Department, University of Medicine and Pharmacy Carol Davila Bucharest, 050474 Bucharest, Romania; gheorghe.dancristian@gmail.com; 2 Electrical Engineering Faculty, University Politehnica of Bucharest, 060042 Bucharest, Romania; marcel.stanciu@upb.ro; 3 Department of Radionuclide Therapy, Institute of Oncology Bucharest, 022328 Bucharest, Romania; ancacvi@gmail.com or; 4 Department of Carcinogenesis and Molecular Biology, Institute of Oncology Bucharest, 022328 Bucharest, Romania

**Keywords:** papillary thyroid cancer, autoimmune thyroiditis, ^131^I, TgAb, TNF-α, TNFR

## Abstract

**Simple Summary:**

Recent evidence shows that autoimmune thyroiditis (AIT) may impair the uptake of radioiodine (^131^I), altering the success of attempted remnant ablation in papillary thyroid cancer (PTC), but the cause is not clear. Finding the mechanisms that govern immune cells during the ^131^I therapy of PTC with concomitant AIT (PTC + AIT) could provide a rationale for these reports. Our study was conducted on female patients admitted for ^131^I therapy. In the PTC group, ^131^I therapy modulates the production of cytokines in situ, increasing the antitumor immune response accordingly. On the contrary, in the presence of chronic inflammation due to AIT, ^131^I therapy amplifies innate immunity, leading to a weaker development of adaptive, specific immunity.

**Abstract:**

Autoimmune thyroiditis (AIT) may impair radioiodine (^131^I) uptake in papillary thyroid cancer (PTC). Finding the mechanisms that govern immune cells during ^131^I therapy of PTC with concomitant AIT (PTC + AIT) could provide a rationale. Our study aimed to evaluate the effects of ^131^I on anti-thyroglobulin antibodies (TgAb), matrix metalloproteinase-9 (MMP-9) and its tissue inhibitor TIMP-1 and tumor necrosis factor-α (TNF-α) and its receptors TNFR1 and TNFR2, in PTC and PTC + AIT patients. Peripheral blood was collected from 56 female patients with PTC and 32 with PTC + AIT before and 4 days after ^131^I (3.7 GBq). The serum levels of TgAb, MMP-9, TIMP-1, TNF-α, TNFR1 and TNFR2 were measured by ELISA. The mean radioactivity of blood samples collected after ^131^I intake was higher in the PTC + AIT group than in PTC (*p* < 0.001). In the PTC + AIT group, TNF-α/TNFR1 and TNF-α/TNFR2 ratios decreased by 0.38-fold and 0.32-fold after ^131^I and were positively correlated with the MMP-9/TIMP-1 ratio (r = 0.48, *p* = 0.005, and r = 0.46, *p* = 0.007). In the PTC group, TNF-α/TNFR1 and TNF-α/TNFR2 ratios increased by 3.17-fold and 3.33-fold and were negatively correlated with the MMP-9/TIMP-1 ratio (r = −0.62, *p* < 0.001 and r = −0.58, *p* < 0.001). Our results demonstrate that TNF-α may exert different antitumor effects in response to ^131^I therapy depending on the patient’s immune profile.

## 1. Introduction

Increasing evidence shows an alarming and unexplained rise in autoimmune conditions over the past 30 years. Autoimmune diseases (AIDs) represent a wide range of disorders manifested by inflammation of organs due to the production of antibodies against self-structures and the cytotoxic action of T cells. For different AIDs, the incidence is rising between 3% and 9% per year [1]. Chronic lymphocytic thyroiditis has shown a 6% increase per year, with a female preponderance of ≥85% [1]. Although current therapies for AIDs aim to inhibit immune cell activation and effector immune pathways, including those activated by cytokines and cytokine receptors [2], in the case of autoimmune thyroiditis (AIT), treatment does not follow this direction. Patients with high titers of thyroid peroxidase antibodies (anti-TPO) or anti-thyroglobulin antibodies (TgAb) but with thyroid function tests in the normal range do not require treatment. On the other hand, AIT features are independent risk factors for PTC development. The association between AIT, papillary thyroid cancer (PTC) and MALT lymphoma is the main topic of case reports [3]. Compared to the general population, patients with AIT have a three-fold higher risk of developing PTC [3,4], accounting for approximately 80% of all thyroid cancers. The coexistence of the two conditions has been reported in many studies [3,4,5,6]. However, regardless of the presence or absence of AIT, the treatment is the same, consisting of thyroidectomy followed by radioactive iodine (^131^I) ablation of the postsurgical thyroid remnant [6]. The radionuclide ^131^I emits beta radiation for intracellular therapy and gamma radiation for defining the presence or absence of remnant thyroid tissue. As all patients have some remnant thyroid tissue after thyroidectomy, strong uptake of ^131^I into the thyroid bed is a prerequisite for remnant ablation. However, due to the toxicity of ^131^I, the focus should be on using the minimum effective dose. Recent evidence shows that concomitant AIT in PTC is associated with a better outcome and lower recurrence rates [4,7]. However, after analyzing the effect of the presence or absence of thyroiditis and different activities of ^131^I administered to a population with differentiated thyroid cancer over a period of three years, Lim et al. reported that concurrent AIT may impair the uptake of ^131^I, altering the success of attempted remnant ablation [8], but the cause is not clear. Finding the mechanisms that govern immune cells during the ^131^I therapy of PTC with concomitant AIT could provide a rationale for these reports.

Recent data have suggested that radiation therapy may modulate antitumor immune responses [9] and induce changes in the local microenvironment, affecting tumor development [10] and not only through “danger” signals that propagate from irradiated to non-irradiated cells [11,12]. The expression of TNF-α is increased after irradiation and may be involved in non-targeted effects, especially in bystander signaling in non-irradiated cells within or in the vicinity of the irradiated area, either via intercellular gap junctions or medium transfer mechanisms along with matrix metalloproteinases (MMPs) and their tissue inhibitors (TIMPs) [13]. It is well known that the tumor necrosis factor-alpha (TNF-α) precursor is cleaved to release soluble TNF-α to increase apoptosis in response to high-dose radiation of tumors [14]. Further, TNF-α can be slowly cleaved from the cell surface by MMPs, producing a bioactive cytokine, soluble TNF-α, which may increase apoptosis through binding to TNF receptor type 1 (TNFR1) [15]. Specifically, our purpose was to evaluate the effects of therapeutic irradiation with ^131^I on the serum levels of TgAb, MMP-9 and its tissue inhibitor TIMP-1 and TNF-α and its receptors TNFR1 and TNFR2, in PTC and PTC associated with AIT (PTC + AIT) patients.

## 2. Materials and Methods

### 2.1. Patients and Study Protocol

Fifty-six female patients with PTC (mean age 43.2 ± 12.7 years) and 32 with PTC associated with AIT (mean age 40.6 ± 12.3 years) were admitted for ^131^I therapy in the Department of Radionuclide Therapy of the Institute of Oncology Bucharest. All PTC with/without AIT patients enrolled in the study were treated with total thyroidectomy, followed by the ablation of residual active tissue in the thyroid bed with an oral dose of ThyroTop^131^, with an average activity around 100 mCi (3.7 GBq) of ^131^I. ^131^I sodium iodide ThyroTop is a radiopharmaceutical and was purchased from Institute of Isotopes Co. Ltd. (IZOTOP), Budapest, Hungary. Indication for ^131^I therapy in this cohort of patients was based on the recommendation from guidelines developed by the European and American Thyroid Associations (ETA and ATA) [16,17] in compliance with safety measures [18]. PTC + AIT patients had positive TgAb antibody titers. The exclusion criteria were as follows: (i) age under 18 years; (ii) acute infection, burning, pulmonary, hepatic or renal impairment; (iii) ongoing treatment with steroidal or non-steroidal anti-inflammatory drugs; (iv) ongoing treatment with antibiotics from the tetracycline group, especially doxycycline (known as MMP inhibitors) [19]. For each enrolled patient, a detailed medical and drug history was obtained. All patients underwent a physical examination and routine laboratory tests. The study protocol consisted of serum TgAb, MMP-9, TIMP-1, TNF-α, TNFR1 and TNFR2 measurements in PTC patients with/without AIT (before and 4 days after the therapeutic dose of ^131^I administration).

The study was conducted following the principles outlined in the Declaration of Helsinki and was approved by the Institute of Oncology Bucharest Medical Ethics Committee (No.15140/10.09.2019). Signed informed consents were obtained from all patients.

### 2.2. Histopathology

Clinical and histopathological data were collected from patients’ medical records. AIT diagnosis is a complex and difficult issue because morphological and serological reports are not always interchangeable, recording different degrees of glandular destruction [20]. The diagnosis criteria for AIT were as follows: (i) background inflammatory infiltrate in the non-neoplastic thyroid (significant numbers of lymphocytes infiltrating the normal thyroid parenchyma away from the neoplastic process; lymphocytes are predominantly T cells and plasma cells—polyclonal); (ii) presence of secondary lymphoid follicles with germinal centers; (iii) atrophic thyroid follicles with minimal colloid, lined by Hürthle cells (metaplastic follicular epithelial cells having abundant eosinophilic cytoplasm and round to oval nuclei with nucleoli); (iv) high titers of TgAb [20,21]. Isolated peri- and intra-tumoral lymphocytic infiltration was not considered as AIT. AIT was preoperatively excluded in all patients in the PTC group. Assessment of the clinical stage was based on the eighth edition of the American Joint Committee on Cancer (AJCC)/TNM cancer staging system (2018) [22]. All the patients enrolled in the study had the classic form of PTC.

### 2.3. Blood Sampling

Fasting blood specimens were collected by venipuncture into BD Vacutainer SST^TM^ II Advance tubes with clot activator (silica particles) right before the start of treatment (^131^I administration) and 4 days after. Blood samples were allowed to clot for 30 min at room temperature before centrifugation for 15 min at 1000× *g*. Then, the serum samples were aliquoted into labeled cryo-vials and stored at −80 °C for further analysis.

It should be noted that blood samples collected 4 days after ^131^I intake were radioactive. A separate procedure for harvesting, transporting, processing and storing radioactive biological samples was prepared. This procedure complies with the rules in force from the European Association of Nuclear Medicine (EANM) guidelines [23] and ISO 15189: 2013/ISO 15190: 2005 (requirements for quality and competence in medical laboratories/requirements for safety in medical laboratories). Blood activity was measured, with a microcurie accuracy, in a dose calibrator (CURIEMENTOR^R^ 3 Isotope Calibrator, PTW Freiburg, Germany). Background count was measured without blood samples before the measurement of activity of each blood sample.

### 2.4. Biomarker Measurements

The ELISA kits required for the quantitative determination of serum MMP-9, TIMP-1, TNF-α, TNFR1 and TNFR2 concentrations were purchased from R&D Systems Inc. (Minneapolis, MN, USA). It is known that contamination can lead to falsely elevated serum levels. Given that MMP-9 and TIMP-1 are present in saliva, additional protective measures (protective mask) were required to prevent serum contamination during testing. Serum samples required a 10-fold dilution for TNFR1 and TNFR2, and 100-fold dilution for MMP-9 and TIMP-1 in different diluents. Polypropylene tubes were also used instead of glass tubes for sample dilution and storage. According to the manufacturer, the values of the intra-assay precision were similar to those of the inter-assay with coefficients of variation ranging from 4.2 to 5.4% for TNF-α, 3.6 to 5.8% for TNFR1, 2.6 to 5.1% for TNFR2, 1.9 to 7.8% for MMP-9 and 3.9 to 5.0 for TIMP-1. The MMP-9/TIMP-1, TNF-α/TNFR1 and TNF-α/TNFR2 ratios were calculated using the following formulas: MMP-9 (ng/mL)/TIMP-1 (ng/mL), TNF-α (pg/mL)/TNFR1 (pg/mL), TNF-α (pg/mL)/TNFR2 (pg/mL). Quantitative determination of serum TgAb concentrations was performed using commercial ELISA kits purchased from IBL International, Hamburg, Germany. The analytical sensitivity or the lower detection limit for TgAb was 10 IU/mL. According to the manufacturer, the values of the intra-assay precision were similar to those of the inter-assay with coefficients of variation ranging from 2.6 to 7.0% for TgAb.

The tests used in the study were 1:1 calibrated against the NIBSC/WHO 2nd International Standard 88/786 for TNF-α [24], WHO standard 65/93 for TgAb [25], the International Tg Reference Material CRM 457 (Community Bureau of Reference, BCR, European Union, Brussels, Belgium) and WHO International Standard for TSH IRP (81/565).

All assays were performed in duplicate, and in such a way as to minimize any effects of repeated freeze–thaw cycles.

### 2.5. Statistics

Patients’ data processing was performed using Microsoft Office Excel 2007 SP2 (including data analysis). Statistical analysis was conducted using Statistica software (version 8.0; StatSoft, Inc., Tulsa, OK, USA). The difference between the serum concentrations of different variables measured in dynamics in the two groups of patients was analyzed by the one-way ANOVA test. The results were expressed as the median (interquartile range (IQR) 25–75%). The distribution of all variables was verified with the Kolmogorov–Smirnov test. The non-parametric Kruskal–Wallis test was used to compare the distribution of continuous variables between different categories for independent samples (PTC group vs. PTC + AIT group). The Wilcoxon test was used for paired samples (PTC group, before vs. after ^131^I intake; PTC + AIT group, before vs. after ^131^I intake). The correlation between investigated biomarkers was assessed using Pearson’s correlation coefficient (r). For all tests, a *p*-value < 0.05 was considered significant.

## 3. Results

### 3.1. Clinicopathological Characteristics

No statistically significant difference was observed between the ages of the female patients enrolled in the two groups (PTC: 43.2 ± 12.7 years vs. PTC + AIT: 40.6 ± 12.3 years, *p* = 0.064).

Analysis of the pathology reports showed that the extrathyroidal extension of PTC was noticed significantly less frequently in patients with AIT: nine patients compared to 34 patients in the group without AIT (*p* = 0.019). Moreover, lymph node metastases were also observed less frequently in patients with AIT: 17 patients compared to 35 patients in the group without AIT, but this finding was not statistically significant (*p* = 0.054).

### 3.2. Blood Sample Radioactivity

The mean radioactivity of blood samples collected 4 days after the administration of the same dose of ^131^I/patient was higher in the group of patients with concomitant AIT than in the group without AIT (1.35 ± 0.09 µCi (49.95 ± 3.33 kBq)/3 mL vs. 0.25 ± 0.07 µCi (9.25 ± 2.59 kBq)/3 mL (*p* < 0.001)).

### 3.3. Effects of ^131^I Irradiation on Analyzed Biomarkers

Biochemical parameters measured in the blood samples collected before the administration of the therapeutic dose of ^131^I (T0) and 4 days after that (T1) from the two groups of patients (PTC and PTC + AIT) are summarized in Table 1. There was no significant difference among the groups in terms of age. In contrast, all analyzed variables were significantly higher in the PTC + AIT patients than in those with PTC at the T0 and T1 time points (*p* < 0.05), except TIMP-1 at the T1 time point (*p* = 0.34). Measurements performed during the T1 time point revealed different ^131^I therapy effects in the two groups, as shown in Table 1. As a first step, ^131^I therapy resulted in a 17% increase in TgAb titer in patients with PTC and concurrent AIT (*p* = 0.02) and a 42% decrease in TgAb titer in patients with PTC (*p* = 0.001). In the PTC + AIT group, the TNF-α/TNFR1 and TNF-α/TNFR2 ratios decreased by 0.38-fold and 0.32-fold after ^131^I (*p* = 0.001 and *p* = 0.003, respectively) and, in the PTC group, the TNF-α/TNFR1 and TNF-α/TNFR2 ratios increased by 3.17-fold and 3.33-fold, respectively (*p* < 0.001).

### 3.4. Correlations

**Figure 1:**
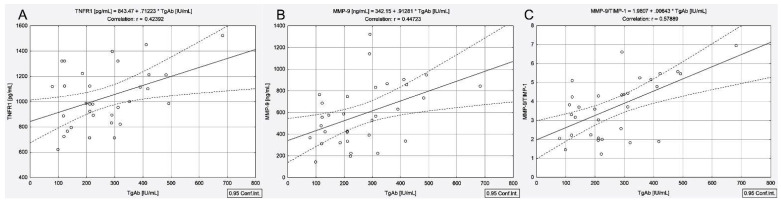
Correlations between serum concentrations of TgAb and TNFR1 (**A**), MMP-9 (**B**), MMP-9/TIMP-1 ratio (**C**) in patients with papillary thyroid cancer associated with autoimmune thyroiditis.

Scatter plots shown in Figure 1 indicate a close relationship between TgAb and TNFR1 (r = 0.42, *p* = 0.016), MMP-9 (r = 0.44, *p* = 0.01) and the MMP-9/TIMP-1 ratio (r = 0.57, *p* < 0.001) in patients with PTC and concomitant AIT.

**Figure 2:**
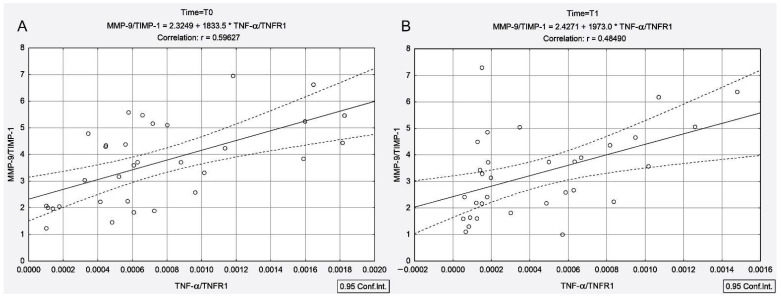
Correlations between MMP-9/TIMP-1 ratio and TNF-α/TNFR1 ratio measured before ^131^I intake (**A**) and at 4 days after ^131^I intake (**B**) in patients with papillary thyroid cancer associated with autoimmune thyroiditis.

**Figure 3:**
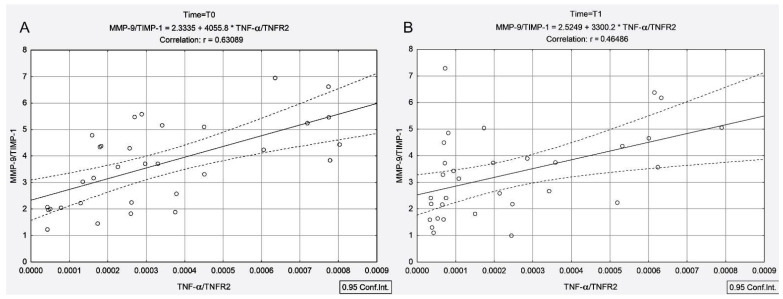
Correlations between MMP-9/TIMP-1 ratio and TNF-α/TNFR2 ratio measured before ^131^I intake (**A**) and at 4 days after ^131^I intake (**B**) in patients with papillary thyroid cancer associated with autoimmune thyroiditis.

Moreover, the TNF-α/TNFR1 and TNF-α/TNFR2 ratios were positively correlated with the MMP-9/TIMP-1 ratio both before and 4 days after ^131^I administration (Figure 2A (T0): r_TNF-α/TNFR1-MMP-9/TIMP-1_ = 0.59, *p* < 0.001; Figure 2B (T1): r_TNF-α/TNFR1-MMP-9/TIMP-1_ = 0.48, *p* = 0.005 and Figure 3A (T0): r_TNF-α/TNFR2-MMP-9/TIMP-1_ = 0.63, *p* < 0.001; Figure 3B (T1): r_TNF-α/TNFR2-MMP-9/TIMP-1_ = 0.46, *p* = 0.007).

**Figure 4:**
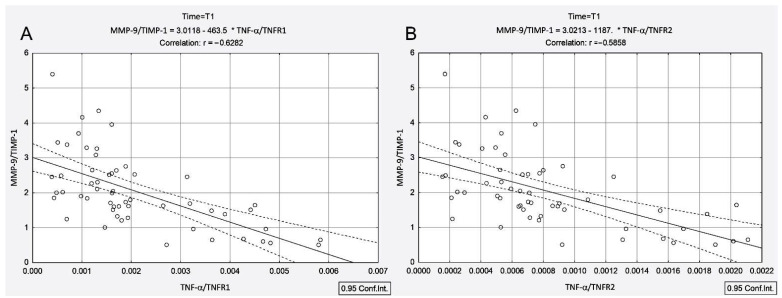
Correlations between MMP-9/TIMP-1 ratio and TNF-α/TNFR1 ratio (**A**) and TNF-α/TNFR2 ratio (**B**) measured at 4 days after ^131^I intake in patients with papillary thyroid cancer.

In contrast to patients with concomitant AIT, in the PTC group, TNF-α/TNFR1 and TNF-α/TNFR2 ratios were negatively correlated with the MMP-9/TIMP-1 ratio only after ^131^I therapy (T1: r_TNF-α/TNFR1-MMP-9/TIMP-1_ = −0.62, *p* < 0.001, r_TNF-α/TNFR2-MMP-9/TIMP-1_ = −0.58, *p* < 0.001), as shown in Figure 4A,B.

## 4. Discussion

^131^I therapy after total thyroidectomy is an integral part of loco-regional therapy for well-differentiated thyroid cancer. ^131^I therapy, as cancer-targeted radiotherapy, can mobilize the host’s immune effector mechanisms that involve both pro-immunogenic and immunosuppressive effects. TgAbs are produced by B lymphocytes, and are considered to be more radiosensitive than T lymphocytes, with a decrease in serum titers after irradiation of 33–90% [26]. In our study, the decline in low-TgAb levels in patients with PTC without AIT was 42%, a percentage that falls within the statistics published to date for B lymphocytes. Unlike the PTC group, in patients with concomitant AIT, ^131^I intake led to a 17% increase in circulating TgAb levels. Our results show that in the presence of high titers of TgAb, the effect of ^131^I is no longer immunosuppressive. The direct effect of a high dose of irradiation is to stimulate the production of B lymphocytes to the detriment of T lymphocytes. As mentioned previously, TgAbs are antibodies produced by B lymphocytes that originate in the bone marrow, mature in the spleen and differentiate into lymph nodes after contact with antigens and T cells and different cytokines. Moreover, the radioactivity of blood samples collected from patients with elevated concentrations of TgAb was higher than that of samples collected from patients with low TgAb levels (1.35 ± 0.09 µCi (49.95 ± 3.33 kBq)/3 mL vs. 0.25 ± 0.07 µCi (9.25 ± 2.59 kBq)/3 mL (*p* < 0.001)). Increased blood radioactivity measured 4 days after the administration of the therapeutic dose of ^131^I indicates a low ^131^I uptake in PTC + AIT patients. Our results are consistent with those obtained by Lim et al. [8], who showed in a retrospective cohort study that the patients with AIT were significantly more likely to have low or no ^131^I uptake, demonstrated on the post-ablation scan.

It is known that ^131^I uptake is influenced by several factors, such as (i) the age of the patient; (ii) the volume of the functional thyroid remnant; (iii) renal clearance; (iv) the iodinated substances (amiodarone, intravenous contrast agents, iodine-containing medicines and preparations); (v) thyroid medications (levothyroxine, liothyronine, anti-thyroid drugs—methimazole, carbimazole); (vi) iodine-containing food. Some of these interfering factors were eliminated through a prior ^131^I low-iodine diet, following the guidelines for ^131^I therapy of differentiated thyroid cancer [16,17]. Regarding the age of the patients enrolled in the study, it should be mentioned that the study groups (PTC and PTC + AIT) matched. Our results showed that PTC + AIT patients had a lower rate of extrathyroidal extension and a non-significant reduction in lymph node metastasis than PTC patients [8,27]. These findings may lead to the idea that the volume of the functional thyroid remnant is lower in the PTC + AIT group than in the PTC group, partially explaining the low ^131^I uptake in patients with concomitant AIT. Our specific aim was to find the mechanisms that govern immune cells during ^131^I therapy of PTC + AIT patients, rather than the success of long-term ablation, which is beyond the scope of this paper. Therefore, we did not review the degree of extrathyroidal uptake seen on the post-ablation. Another factor that could influence the ^131^I uptake and should be considered could be the increased titers of TgAb. Detailed studies are needed to debate this subject.

When ^131^I accumulates in the remnant thyroid tissue, its decay results in thyroid cancer cells’ high energy deposition and minimal irradiation of the surrounding normal tissues. The expression of TNF-α and TIMP-1 is increased after irradiation [12,13,28] and can be involved in the ^131^I-induced bystander effect. Similar to the previously mentioned studies [12,13,28], our results showed that ^131^I intake led to a 3.55-fold increase in TNF-α and a 1.19-fold in TIMP-1 in patients without AIT. Moreover, the involvement of TNF-α and the MMP-9/TIMP-1 complex in the radiation-induced non-targeted effects has been demonstrated by the highly statistically significant correlations measured between TNF-α and TIMP-1 (r = 0.68, *p* < 0.001) and TNF-α and the MMP-9/TIMP-1 ratio (r = −0.66, *p* < 0.001). Instead, in the presence of AIT, TNF-α decreased with a median value of 31% and TIMP-1 remained unchanged, with the two parameters not being statistically correlated. TNF-α has been shown to have a dual role with opposite effects on tumor development: at low concentrations, TNF-α promotes angiogenesis and metastasis of tumor cells, and at high levels has antitumor effects [29]. It seems that TNF-α and the MMP-9/TIMP-1 complex work together with ^131^I targeted therapy. The involvement of the TNF-α and MMP-9/TIMP-1 complex in radiation-induced non-targeted effects led to the inhibition of tumor angiogenesis, highlighted by the 0.34-fold decrease in the MMP-9/TIMP-1 ratio in patients without AIT, which was only 0.8-fold in patients with coexisting AIT.

In response to high-dose irradiation, in our case, irradiation with an average dose of 100 mCi (3.7 GBq)/patient, TNF-α precursor is slowly cleaved from the cell surface by MMPs, producing soluble TNF-α, which can increase apoptosis by binding to TNFR1 [15]. TNFR1 signaling contributes to the pathological processes of autoimmune disorders, promoting, on the one side, inflammation in AIT [30] and, on the other side, being a key factor in the tumor microenvironment, the ^131^I apoptotic response. As a confirmation, our results showed a statistically significant correlation between TgAb and TNFR1 (r = 0.42, *p* = 0.016), MMP-9 (r = 0.44, *p* = 0.01) and the MMP-9/TIMP-1 ratio (r = 0.57, *p* < 0.001) in patients with PTC and concomitant AIT. Conversely, TNFR2 is mainly limited to immune and endothelial cells, supporting the regulatory T cells’ function [31].

In the PTC group, ^131^I intake led to an increase in the TNF-α/TNFR1 and TNF-α/TNFR2 ratios (by 3.17-fold and 3.33-fold), with these increases being negatively correlated with the MMP-9/TIMP-1 ratio (r_TNF-α/TNFR1-MMP-9/TIMP-1_ = −0.62, *p* < 0.001, r_TNF-α/TNFR2-MMP-9/TIMP-1_ = −0.58, *p* < 0.001) (Figure 4). Unlike the PTC group, in the presence of concomitant AIT, ^131^I intake led to a decline in the TNF-α/TNFR1 and TNF-α/TNFR2 ratios (by 0.38-fold and 0.32-fold, respectively), with these decreases being positively correlated with the MMP-9/TIMP-1 ratio (r_TNF-α/TNFR1-MMP-9/TIMP-1_ = 0.48, *p* = 0.005, r_TNF-α/TNFR2-MMP-9/TIMP-1_ = 0.46, *p* = 0.007) (Figure 2B and Figure 3B). It should be noted that prior to irradiation in the PTC + AIT group, there was a strong correlation between TNF-α/TNFR1, TNF-α/TNFR2 and MMP-9/TIMP-1 (r_TNF-α/TNFR1-MMP-9/TIMP-1_ = 0.59, *p* < 0.001, r_TNF-α/TNFR2-MMP-9/TIMP-1_ = 0.63, *p* < 0.001) (Figure 3A,B), while in the group without AIT, there was no correlation between these parameters.

The most apparent weakness of our study is related to the small number of patients included. Despite the small sample size, the two study groups matched in terms of number, gender distribution and age.

Considering the immune response to ^131^I therapy, the two groups of patients can be seen as mirror images of each other. In this regard, in patients with PTC, ^131^I therapy modulates the production of cytokines in situ, increasing the antitumor immune response accordingly. On the contrary, in the context of the existence of chronic inflammation due to AIT, ^131^I therapy amplifies innate immunity and leads to a weaker development of adaptive, specific immunity.

Elevated TNF-α/TNFR1 and TNF-α/TNFR2 ratios indicate a decline in disease activity after ^131^I therapy, which is more pronounced in PTC than in PTC + AIT, suggesting that suppression of TNFR1 and TNFR2 or increased production of TNF-α is required to initiate remission of cancer. In PTC patients, ^131^I therapy almost halved the imbalance between MMP-9 and TIMP-1 and this decrease may reduce tumor cell viability and migratory potential.

Our results demonstrate that the same mediator, in this case, TNF-α, may exert different antitumor effects in response to ^131^I therapy depending on the patient’s immune profile and, therefore, in a clinical setting, may represent a double-edged sword. Certainly, more information is needed before drawing firm conclusions about the non-targeted effects induced by ^131^I in PTC patients with/without AIT.

## Figures and Tables

**Table 1:** Biochemical parameters in the papillary thyroid cancer and papillary thyroid cancer associated with Hashimoto thyroiditis patients before and 4 days after radioiodine therapy.

Variables	PTC		PTC + AIT	
	*n* = 56		*n* = 32	
	T0 ^c^	T1 ^d^	T0 ^c^	T1 ^d^
Age (years) ^a^	43.2 ± 12.7	43.2 ± 12.7	40.6 ± 12.3	40.6 ± 12.3
TgAb (IU/mL) ^b^	7.9 (6.5–9.4)	5.2 (3.5–7.2)	222.7 (138.8–335.9)	292.0 (194.0–354.4)
TNF-α (pg/mL) ^b^	0.37 (0.20–0.55)	1.3 (0.9–2.4)	0.65 (0.35–0.90)	0.30 (0.20–0.90)
TNFR1 (pg/mL) ^b^	739.9 (664.5–968.6)	796.2 (700.4–997.1)	986.4 (858.8–1212.4)	1326.9 (1051–1521.5)
TNFR2 (pg/mL) ^b^	1942.9 (1527.3–2301.9)	2102.9 (1626.7–2498.6)	2318.5 (2116.8–2610.3)	2542.8 (2234.7–2794.4)
MMP-9 (ng/mL) ^b^	395.6 (317.4–555.5)	300.6 (222.9–376.9)	561.7 (351.6–798.4)	509.4 (331.4–660.5)
TIMP-1 (ng/mL) ^b^	133.2 (120.5–155.4)	152.7 (126.5–168.7)	154.1 (132.4–176.3)	152.4 (141.2–180.7)
TNF-α/TNFR1 ^b^	4.5 × 10^−4^ (3 × 10^−4^–7.5 × 10^−4^)	16 × 10^−4^ (4 × 10^−4^–58 × 10^−4^)	6.1 × 10^−4^ (4.3 × 10^−4^–10 × 10^−4^)	2.5 × 10^−4^ (1.3 × 10^−4^–6.5 × 10^−4^)
TNF-α/TNFR2 ^b^	2 × 10^−4^ (1.3 × 10^−4^–2.7 × 10^−4^)	7 × 10^−4^ (5.1 × 10^−4^–9.3 × 10^−4^)	2.7 × 10^−4^ (1.6 × 10^−4^–4.5 × 10^−4^)	1.3 × 10^−4^ (0.7 × 10^−4^–3.5 × 10^−4^)
MMP-9/TIMP-1 ^b^	3.1 (2.4–3.7)	1.8 (1.3–2.5)	3.7 (2.1–4.9)	3.2 (2.2–4.4)

MMP-9, matrix metalloproteinase-9; PTC, papillary thyroid cancer; PTC + AIT, papillary thyroid cancer associated with autoimmune thyroiditis; TgAb, anti-thyroglobulin antibodies; TIMP-1, tissue inhibitor of metalloproteinase-1; TNF-α, tumor necrosis factor-α; TNFR1, TNF receptor type 1; TNFR2, TNF receptor type 2. ^a^ Mean ± standard deviation. ^b^ Data are expressed as median values and interquartile ranges (25–75%).^c^ PTC group (T0) vs. PTC + AIT group (T0): *p* < 0.05. ^d^ PTC group (T1) vs. PTC + AIT group (T1): *p* < 0.05 (except TIMP-1 with no statistical significance).

## Data Availability

The data presented in this study are available on request from the corresponding author.
